# Considerations for Cannabis Use to Treat Pain in Sickle Cell Disease

**DOI:** 10.3390/jcm9123902

**Published:** 2020-12-01

**Authors:** Donovan A. Argueta, Anupam Aich, Fjolla Muqolli, Hemanth Cherukury, Varun Sagi, Nicholas V. DiPatrizio, Kalpna Gupta

**Affiliations:** 1 Hematology/Oncology, Department of Medicine, University of California, Irvine, CA 92868, USA; daarguet@hs.uci.edu (D.A.A.); anupam.rajon@gmail.com (A.A.); fmuqolli@hs.uci.edu (F.M.); hemu.cherukury@gmail.com (H.C.); 2 Department of Hematology, Oncology, and Transplantation, University of Minnesota, Twin Cities, MN 55455, USA; sagix002@umn.edu; 3 Division of Biomedical Sciences, School of Medicine, University of California, Riverside, CA 92521, USA; ndipatri@medsch.ucr.edu; 4 Southern California Institute for Research and Education, Long Beach VA Medical Center, Long Beach, CA 90822, USA

**Keywords:** cannabis, cannabinoid, pain, sickle cell disease, vaso-occlusive crises

## Abstract

Pain in Sickle Cell Disease (SCD) is a major comorbidity and unique with acute pain due to recurrent and episodic vaso-occlusive crises as well as chronic pain, which can span an individual’s entire life. Opioids are the mainstay treatment for pain in SCD. Due to recent health crises raised by adverse effects including deaths from opioid use, pain management in SCD is adversely affected. Cannabis and its products are most widely used for pain in multiple conditions and also by patients with SCD on their own. With the availability of “Medical Cannabis” and approval to use cannabis as medicine across majority of States in the United States as well as over-the-counter preparations, cannabis products are being used increasingly for SCD. The reliability of many of these products remains questionable, which poses a major health risk to the vulnerable individuals seeking pain relief. Therefore, this review provides up to date insights into available categories of cannabis-based treatment strategies, their mechanism of action and pre-clinical and clinical outcomes in SCD. It provides evidence for the benefits and risks of cannabis use in SCD and cautions about the unreliable and unvalidated products that may be adulterated with life-threatening non-cannabis compounds.

## 1. Introduction

Pain in Sickle Cell Disease (SCD) is a major comorbidity and unique [1]. It can arise from acute, unpredictable episodes of vaso-occlusive crises (VOCs) that may begin in infancy and continue throughout life [1]. Additionally, chronic pain, with or without acute pain crises, occurs in a large proportion of SCD patients. In a study spanning just over 31,000 patient days, 232 adult SCD patients experienced chronic pain on more than 54% of total days [2]. Acute, painful VOCs are a characteristic feature of SCD that require hospitalization, impair quality of life, and impact patient survival [3]. Both acute as well as chronic pain are life disabling. Opioids are the mainstay for pain management in SCD, but high doses of opioids are required and are associated with adverse effects including pruritus, tolerance and opioid-induced hyperalgesia (OIH) [4,5]. Rates of opioid overdose are low in patients with SCD and have not shifted with the opioid epidemic [6,7]. Cannabinoids have been widely considered for treating pain to meet the demand for alternative pain management therapies [8].

Evidence of human use of the *Cannabis sativa* L. plant in rituals and medicine dates back millennia [9]. In 1970, cannabis was classified as a Schedule 1 drug in the United States (U.S.), and it was deemed to have no known medical use and a high potential for abuse [10]. Despite the prohibition of cannabis in the U.S. and many European countries, there has been steady progress in studying its constituents for their beneficial effects in many conditions [11,12,13]. An analysis of cannabis use among people using opioids for chronic pain management reported greater pain relief with cannabis than with opioids used alone in a national survey of adults in the U.S. [14]. In addition, emerging evidence suggests that prescriptions for opioids and deaths attributed to opioid use have declined in states that have made medicinal cannabis legal [15,16,17].

Sickle patients often report use of cannabis to manage pain [18,19,20]. It will be an understatement to say that the opioid epidemic and Centres for Disease Control guidelines on opioid use in 2016 have added another hurdle to pain management in SCD because pain treatment for both persistent chronic and acute VOC pain is reliant on opioids [7,21,22]. Surveys conducted on residents involved in pain management of SCD suggest that potential for opioid tolerance and dependence pose a major hurdle in prescribing opioids [22]. Similarly, adults with SCD reported using cannabis due to increased stigmatization for seeking opioids for pain, recent inadequate opioid dosing by the prescribers, and lack of alternatives through healthcare providers [21]. Similar challenges in opioid prescribing for pain management among providers and patients have been disruptive to lives of patients living with chronic pain in other conditions as well [23,24,25]. Thus, inadequate pain management due to fear of opioid prescribing and dosing and stigmatization for continued requirement of opioids in SCD contribute to use of cannabis and related easily available products by the patients to find pain relief. On the other hand, it provides a compelling reason to evaluate the potential of cannabis and its many non-toxic products for the potential to treat sickle pain. Clinical pain management with opioids is presented in another review in this Special Issue and is thus not discussed in detail herein.

Cannabinoids represent a promising alternative due to their tolerability and pre-clinical evidence for their efficacy in attenuating chronic and acute hyperalgesia in SCD [26,27,28,29]. A recent prospective clinical trial of vaporized cannabis use in SCD also shows promise for cannabinoid use without any significant adverse events [30]. Hence, we discuss the mechanism-based understanding of using cannabinoids to treat pain based on pre-clinical and clinical observations in SCD. More importantly, we critically review the benefits and risks of cannabis use in the current environment flooded with “Medical Cannabis” and uncontrolled availability of cannabis products over the counter. We have used the word cannabis when cannabis has been used and cannabinoids as a general term for products derived from cannabis or synthetic cannabinoids.

## 2. Cannabinoids and Their Receptors

Cannabinoids comprise a broad class of plant-derived, synthetic, and endogenously produced compounds that act via cannabinoid receptors 1 and 2 (CB_1_R and CB_2_R, respectively) [31,32,33,34] and possibly others [35,36]. The major plant-derived cannabinoids from *Cannabis sativa* L. are Δ^9^-tetrahydrocannabinol (THC) and Cannabidiol (CBD) [37,38]. There also exists a class of endogenously produced cannabinoids, dubbed endocannabinoids (eCBs); the major eCBs are anandamide and 2-arachidonoyl-*sn*-glycerol (2-AG), which are lipid-based signaling molecules that are produced on-demand [39]. There has been a cascade of synthetic cannabinoids that act with higher potency than plant-derived and endogenous cannabinoids, which are invaluable research tools though many have potential for abuse [33]. Cannabinoids exert their effects through interactions with the eCB system.

The eCB system comprises the cannabinoid receptors, their endogenous ligands—the eCBs—and corresponding biosynthetic and degradative enzymes [39]. Emerging strategies for leveraging the eCB system in various models of pain include targeting the enzymes responsible for production and breakdown of eCBs [40,41]. The intoxicating effects of THC are mediated through activation of CB_1_R, which are concentrated in the central nervous system (CNS) and are also expressed diffusely throughout the mammalian body [42]. CB_1_R activation has been shown to modulate pain, appetite, cognition, emesis, reward (addiction), neuroexcitability, balance, thermoregulation and motor function [43,44]. CB_2_R are expressed primarily on immune cells and display roles in regulating responses to pain, immune challenge, inflammation, and cell proliferation [28,45,46]. CBD has been suggested to act via modulation of CB_1_R and/or other mechanisms, and we have previously discussed CBD for use in chronic pain [29].

## 3. Mechanisms of Pain in Sickle Cell Disease

SCD originates from a single point mutation of the beta globin gene of hemoglobin that leads to rigid sickle-shaped red blood cells (RBCs) in a deoxygenated state [47]. The biological underpinnings of pain in SCD remain poorly understood. Pain in SCD may be a direct consequence of avascular necrosis or lower limb ulcers [48,49]. It is known that sickle RBCs cause vaso-occlusion leading to impaired blood and oxygen supply to the organs resulting in end-organ damage and acute, unpredictable and recurrent episodes of pain [1,50,51]. Inflammation, endothelial activation, oxidative stress, ischemia/reperfusion injury, and hemolysis underlie sickle pathobiology, which are further enhanced in the wake of VOCs [52]. The underlying mechanism for how vaso-occlusion leads to pain remains incompletely understood.

### 3.1. Mechanisms Involving the Nervous System

In the last decade, strong pre-clinical findings have characterized chronic pain and the underlying key mechanisms that cause it [53,54,55]. These include neurogenic and neuro inflammation [26,28,56], activation of transient receptor potential vanilloid 1 (TRPV1) [26,57], peripheral nerve damage [26], peripheral and central sensitization [58,59], spinal glial activation [60,61], increased blood–brain barrier permeability [62,63], mast cell activation [56], and Purkinje cell damage in the cerebellum [64,65]. Neuroinflammation demonstrated with increased circulating substance P (SP) [66,67,68] and glial fibrillary acidic protein (GFAP) [69] and central sensitization have also been observed clinically. Dorsal horn neurons in preclinical sickle models also demonstrated higher excitability in concert with activation of signaling pathways that promote neuronal excitability [58] with increased GFAP-expressing astroglial cells [60] and microglial activation [61]. Therefore, humanized mouse models of SCD have provided mechanistic insights that mimic key features and mechanisms of pain observed clinically.

### 3.2. Neuroimmune Mechanisms

**Figure 1:**
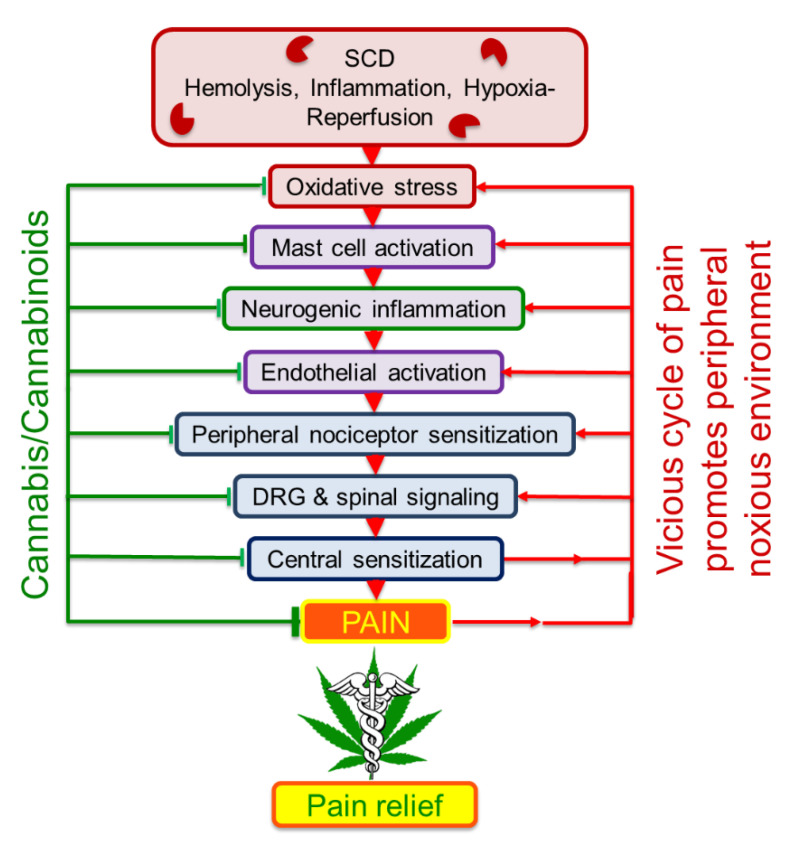
**Cannabinoids ameliorate activation of peripheral and central mediators of sickle pain**. In sickle cell disease ongoing hemolysis, inflammation, oxidative stress, mast cell activation, neurogenic inflammation, hypoxia/reperfusion injury and endothelial activation provide a noxious microenvironment that leads to nociceptor activation in the periphery as well as the central nervous system (CNS). Continued neuronal activation in the dorsal root ganglia leads to the amplification and transmission of action potentials to the spinal second order neurons causing hypersensitization resulting in central sensitization and pain refractory to treatment. Continued sensitization causes an antidromic release of substances from the CNS to the periphery causing neuroinflammation, which in turn lead to sensitization of peripheral nerve fibers and transmission of action potentials, thus leading to a vicious cycle of peripheral and central sensitization and pain. Cananbinoids have the potential to inhibit oxidative stress, inflammation, neuroinflammation and peripheral/central sensitization, thereby ameliorating the underlying pathobiology of sickle cell disease that may initiate pain and also by directly inhibiting the neuronal activity, leading to amelioration of pain.

The discovery of pain mediation by mast cells was the foremost demonstration of neuroimmune interactions affecting sickle pain [56]. Inflammation and neuroinflammation arising from increased glial, neutrophil, monocyte, mast cell and neural activation and neurogenic inflammation underlie nerve injury leading to neuropathic pain, which may present non-uniformly in sickle patients as suggested by quantitative sensory testing (QST) [70,71,72,73,74,75]. Hypersensitivity and lower threshold to mechanical and thermal stimuli on QST in patients with SCD may be due to injury to the peripheral and/or central nervous system, evoked by neuroinflammatory substances such as SP [72,76,77,78]. Sickle patients have higher plasma levels of SP, tryptase and GFAP, markers of neuroinflammation [66,67,69,79]. Tryptase is released from mast cell activation and sickle patients with acute myeloid leukemia benefited from mast cell inhibitor imatinib treatment exhibited by amelioration of VOC [80,81,82]. In our preclinical studies, inhibiting mast cell activation with imatinib elicited significant analgesic response along with reduced expression of SP/calcitonin gene-related peptide (CGRP), systemic inflammation, neurogenic inflammation and neuroinflammation [56]. Our results indicated that activated mast cells in sickle microenvironment release tryptase eliciting SP and CGRP from peripheral nerve endings. Vasoactive SP and CGRP lead to neurogenic inflammation by stimulating vascular permeability in sickle mice [56]. Persistent mast cell activation in a feed-forward loop orchestrated by SP and other inflammatory mediators may contribute to the sustained sensitization of the peripheral nociceptors and consequently spinal neurons. Cannabinoids have been shown to inhibit mast cell activation [28,56], and therefore have the potential to ameliorate sickle pain and VOC (Figure 1).

## 4. Effect of Cannabinoids on Sickle Pain and Sickle Pathobiology

We found that CP55,940, a high-affinity CB_1_R and CB_2_R agonist, significantly reduced chronic and hypoxia-reoxygenation (HR)-evoked hyperalgesia, which mimics VOC pain, in transgenic sickle mice [26,27,28]. CP55,940 also ameliorated features of sickle pain including increased sensitivity to touch and temperature extremes, spontaneous musculoskeletal/deep tissue hyperalgesia, and HR-evoked hyperalgesia in sickle mice [26,27,28]. Pre-clinical studies suggest that cannabinoids, including the eCBs anandamide and 2-AG, may ameliorate pain and address the underlying pathophysiologic changes in SCD. We found that URB597, which blocks the hydrolysis of anandamide, reduced c-fiber nociceptor sensitization and associated hyperalgesia in a preclinical sickle model in a CB_1_R-specific manner [59]. Moreover, the CB_1_R agonist arachidonyl-2′-chloroethylamide (ACEA) and CB_2_R agonist JWH-133 both attenuated deep tissue hyperalgesia, but only ACEA reduced HR-evoked mechanical and thermal hyperalgesia [28]. While CB_1_Rs are critical for analgesia, non-intoxicating cannabis-derived CBD and the CB_2_R pathway have been demonstrated to ameliorate pain in part via TRPV1 and at the supraspinal level in animal models of neuropathic pain [83,84]. Pain is also accompanied by stress in SCD. Stress-induced neuroinflammation was significantly attenuated in wild-type mice treated with JWH-133 and mice overexpressing CB_2_R, but not in CB_2_R-knockout mice [85]. Therefore, CB_2_R agonists augment CB_1_R analgesia in sickle pain, and both may be required to achieve effects similar to those from whole plant-based compounds found in cannabis.

Cannabinoids attenuate inflammation, leukocyte trafficking and adhesion, mast cell activation, oxidative stress, ischemia/reperfusion injury and neurogenic inflammation via CB_1_Rs and CB_2_Rs (Figure 1). All these phenomena exacerbate pain and may underlie clinical features of SCD including impaired wound healing, renal damage, and retinopathy [4,56,86]. Our finding that CP55,940 reduces hyperalgesia was associated with reduced mast cell activation, leukocyte counts and neurogenic inflammation [28]. Severe inflammation in SCD is characterized by elevated cytokines, pro-inflammatory and vasoactive neuropeptides, in both humans and sickle mice [4,56,67,86,87,88]. Microglial activation with significantly higher cytokine levels, toll-like receptor 4 (TLR4) expression and Stat3 phosphorylation in sickle mice spinal cords suggest a central inflammatory milieu [60]. In animal models of diverse diseases, CB_2_R was found to mediate the anti-inflammatory effect of cannabinoids such as CBD, HU210, and WIN55,212-2, both peripherally and centrally [89]. THC exhibits an anti-inflammatory effect that is mediated primarily through CB_1_Rs; however, CB_2_Rs do appear to play a critical role in regulating inflammation in most cellular and animal studies. Therefore, cannabinoids have the potential to target many mechanisms underlying pain in SCD and other comorbidities.

Inflammation, hemolysis, and cell-free hemoglobin in the hypoxic sickle microenvironment cause oxidative stress in SCD [90]. WIN55,212-2, CP55,940 and anandamide exert a protective effect on quinolinic acid-induced mitochondrial dysfunction, reactive oxygen species (ROS) formation and lipid peroxidation in rat striated cultured cells and rat brain synaptosomes [91]. Importantly, in parkin-null, human tau overexpressing (PK-/-/TauVLW) mice, a model of complex neurodegenerative disease, short-term Sativex (Nabiximols, 1:1, THC:CBD preparation) administration significantly reduced intraneuronal monoamine oxidase-related free radicals, increased the ratio of reduced/oxidized glutathione, and improved behavioral and pathological abnormality [92]. Consistent with these observations in other pathologies, cannabinoids may also reduce oxidative stress and pain in SCD.

Erythrocyte adhesion, nitric oxide depletion, hemolysis, oxidative stress and inflammation accompany endothelial dysfunction in SCD [93,94]. Endothelial activation causes upregulation of adhesion molecules including selectins, vascular cell adhesion molecule and intercellular adhesion molecule 1, which exacerbate vaso-occlusion and end-organ damage [47]. CB_1_R and CB_2_R are widely expressed on vascular smooth muscle cells and endothelium [95]. Both receptors have been widely studied in vascular relaxation and activation of ion channels including potassium, calcium and TRPVs. Antagonistic roles are demonstrated in different settings and disease states with respect to CB_1_R and/or CB_2_R. Thus, it is likely that cannabinoids influence endothelial function in a sickle-specific microenvironment.

## 5. Clinical Studies on the Effect of Cannabinoids on Pain

Cannabis and cannabinoids have been evaluated clinically for their analgesic potential in various disease states, and recently these findings have been described in a systematic review [96]. Studies indicate that smoked cannabis may provide analgesic support in chronic and neuropathic pain, but smoking is associated with its own risks and pathologies; thus, other formulations and routes of administration are also being investigated [97,98,99]. To date, several double-blind placebo-controlled studies have been completed to evaluate the safety and efficacy of oral THC and/or Sativex which delivers a controlled dose of 2.7 mg THC and 2.5 mg CBD per spray [100]. Sativex has also been tested in several pain contexts, including cancer, chronic abdominal pain, multiple sclerosis, brachial plexus injury, and diabetic neuropathy. In a study of chronic abdominal pain, oral THC did not reduce measures of pain, but was well-tolerated and absorbed over a 2-month period [101]. In contrast, Sativex was effective at providing sustained relief of central neuropathic pain in patients with multiple sclerosis on fixed and self-titrating schedules compared to patients receiving placebo [102,103]. Moreover, Sativex improved pain at targeted responder levels and significantly improved sleep in difficult-to-treat neuropathic pain arising from brachial plexus avulsion and allodynia-characterized neuropathic pain [104,105]. The latter study was followed-up with a 52-week open-label trial in which pain relief was maintained without dose increase or toxicity [106]. While promising, these studies must be evaluated critically due to their potential for biases related to sampling [107].

Another growing concern is the safety of approaches to alter endocannabinoids, which was most notable with the failed study involving the fatty acid amide hydrolase (FAAH) inhibitor BIA 10-2474 [108]. The study was terminated following the death of a patient and irreparable side-effects in other participants. In retrospect, the compound was not as selective of an inhibitor as it was previously believed to be, and early signs of toxicity in pre-clinical studies went ignored [109]. This instance highlights the need for careful, well-controlled pre-clinical studies before undertaking clinical trials.

To date, several other clinical studies involving cannabis, THC preparations, and/or Sativex have been completed in patients with chronic pain arising from various diseases. Results from these studies indicate no effect to mild effect at reducing chronic pain, improving sleep quality, and improving patient-reported quality of life. Side-effects from these studies are also limited, and it appears that low doses are well-tolerated. The results from these studies, however, have not undergone peer review, and thus must be heavily scrutinized before any recommendations can be made. The identifiers for the aforementioned studies follow: NCT01606202, NCT00713817, NCT00710424, NCT01606176, NCT01262651, and NCT00241579.

## 6. Clinical Use of Cannabis in Sickle Cell Disease

In SCD, limited data are available on pain management with cannabis. Moreover, most are surveys, patient’s self-reports, retrospective analysis, detection on urine screening, use of multiple non-prescribed drugs and most often not adjusted for gender, disease severity, opioid use, other comorbidities contributing to pain such as leg ulcers or avascular necrosis and prescription medications for many comorbidities. However, emerging prospective well-designed studies have started providing unbiased insights relevant to cannabis use in SCD. We briefly describe salient findings from different studies on cannabis use in SCD (see Table 1).

### 6.1. Fewer Admissions in Cannabis Users with Pain in SCD

Increased access to medicinal cannabis has also shifted open use in SCD patients, with studies reporting greater disease severity and decreased in-patient hospitalizations in patients receiving medicinal cannabis [110,111,112]. A cross-sectional study of adults with SCD (aged 18 and older) was performed at the Yale New Haven Hospital, based on patient reported outcomes for pain and health-related quality of life (HRQoL) questionnaire using the Adult Sickle Cell Quality of life Measurement Information System (ASCQ-Me) to assess VOC pain frequency/severity and impact of pain and Patient-Reported Outcomes Measurement Information System (PROMIS) for qualitative assessment of nociceptive and neuropathic pain [111]. The effect of cannabis on baseline pain and acute pain HRQoL outcomes was examined factoring in for SCD genotype, disease severity, age, gender, genotype, hydroxyurea (HU) use, oral morphine equivalents and transfusions, etc. Approximately 20% of SCD subjects reported using cannabis daily compared to 55% non-users and others who used weekly, monthly or in between. Daily users reported significantly higher pain episode severity scores than non-users (*p* = 0.02). However, propensity matched with variables on pain outcomes showed that daily cannabis users reported fewer annual ER visits and annual admissions. Matched for pain impact score for daily pain with other aforesaid variables, daily users had 1.8 and 1.2 fewer annual admissions and ER visits. Similarly, using daily opioids dispensed as a measure of pain matched for other variables showed daily users had 2.5 and 1.5 fewer annual admissions and ER visits compared with others. Since daily users had more severe pain crises, it is inferred that daily use is associated with higher severity of pain crises and that comparisons need to factor in the pain severity and account for other factors such as ability to tolerate pain better.

### 6.2. Prospective Trial Shows Vaporized Cannabis Is Well Tolerated in Adults with SCD

A pilot study performed by our group investigated the analgesic potential of vaporized cannabis in SCD patients (NCT01771731) [30]. Twenty-three patients with SCD-related chronic pain receiving opioids completed a randomized double-blind placebo-controlled crossover trial, inhaling vaporized cannabis or placebo during two separate five-day inpatient sessions that were separated by a 30-day washout period. Vapors were collected in-house by vaporizing cannabis containing 4.4% THC and 4.9% CBD, obtained from the National Institute on Drug Abuse. The crossover design allowed for each patient to serve as their own control. Pain was assessed throughout each treatment period along with pain interference measures. The crossover-pain difference between cannabis and placebo treatment was negative for each treatment day indicating a decrease in pain with cannabis treatment; however, this decrease was not statistically significant. Additionally, pain levels were generally lower in patients given cannabis when compared to those given placebo, but this difference was also not statistically significant. As each five-day study period progressed, patients given cannabis reported that pain interfered less with activities, including walking and sleeping, with a statistically significant decrease in interference with mood. Importantly, this study showed that vaporized cannabis is well-tolerated and significantly improves “mood” in SCD patients with chronic pain. The lack of significant adverse effects in this study encourages further investigation into the use of cannabis-based interventions including CBD to treat chronic SCD pain in prospective trials with a larger cohort over a longer duration [30].

### 6.3. Self-Management of Pain with Cannabis

Questionnaire-based approaches have provided insight into the prevalence of cannabis use in the SCD community, and these studies have given first-hand accounts of the patients’ perceived benefits and motivations for seeking cannabis [21,119]. A 2018 survey of 58 patients living with SCD revealed cannabis use in 42% of respondents [20]. The majority of these individuals reported medicinal purposes, though some indicated recreational use of cannabis. The self-reported use further indicates the need to study cannabis to understand its potential risks versus benefits.

An anonymous questionnaire study of Sickle Cell patients in the United Kingdom included 31 patients who had used cannabis and 51 patients who had never used it, although this group represents only 34% of individuals that qualified for the study and chose to participate [18]. Responses indicated that cannabis users had more frequent and more severe episodes of pain, but many of the users indicated that cannabis was an attempt at managing their pain. Cannabis users reported improvement in mood (35%), reduced use of painkillers (42%), improvement in feelings of anxiety and depression (52%), and improvement in sleep (61%) [18]. In addition, 58% of respondents indicated an interest in participating in future clinical trials for the study of cannabis in SCD pain management [18]. This questionnaire-based study underscores the attractiveness of cannabis as a means of self-medicating for pain, but this also presents another potential concern; to circumvent the prohibition of cannabis, individuals may resort to use of unregulated, potentially dangerous synthetic cannabinoid analogs.

### 6.4. Juvenile Use of Cannabis in SCD

Neuropathic pain is disabling and impairs the HRQoL in adolescents as well. In a preliminary study of 12 adolescents with mean age of 15 years, with 75% females and 83% of subjects on hydroxyurea, higher PainDETECT scores were significantly associated with lower PedsQL (indicator of HRQoL) scores [121]. Cannabis use in teenagers with SCD and cystic fibrosis is prevalent, although to a lower extent than their peers [120], which may be due to the perception of cannabis use associating with worse self-care, more stress, and more distress [113,114].

### 6.5. Urine Screening Revealed Use of Cannabis and Other Drugs

A 2017 retrospective analysis of patients with SCD indicated that patients using cannabis, confirmed by urinalysis, had higher frequency of VOCs [115]. This study comprised 37 SCD patients that tested positive for a THC metabolite and 35 that tested negative. Notably, patients who tested positive admitted to smoking cannabis as their route of administration. Additionally, cannabis users had significantly higher use of benzodiazepine, cocaine, and phencyclidine compared to non-users. The use of other illicit compounds may potentiate the negative effects associated with cannabis use in this retrospective analysis. In addition, cannabis users had significantly fewer visits to the clinic and increased hospital admissions compared to non-users; the lack of regular treatment and increased disease severity may also represent contributing variables that are difficult to control. Priapism, mortality, and other SCD co-morbidities were not different between groups [115]. Opioid-induced hyperalgesia and tolerance to specific opioids has been suggested to lead to cannabinoid and phencyclidine use in an individual with SCD [122]. The reason for using cannabis in this patient was that pain relief was inadequate with Percocet. After switching to morphine, his urine showed the presence of phencyclidine, which provided him better pain relief than morphine. These studies highlight the inadequacy and changing needs of patients with persistent and/or VOC pain in SCD leading to cannabis use and perhaps of other drugs that they can get to find relief.

### 6.6. Cannabis Use and Other Comorbidities of SCD

In a retrospective observational study on 9350 patients 18 years and older admitted for acute ischemic stroke (AIS) who underwent urine drug screening screening, 18% tested positive for cannabis [123]. Among cannabis users unadjusted risk ratio showed a 50% decrease in risk of AIS. However, upon adjusting for SCD, cardiovascular disease, diabetes, cigarette smoking, ethnicity, age, race, etc., the effect was lost. Many limitations in this study included dosage and duration of cannabis use, but it does not show any adverse effect of cannabis on AIS. These findings are important because stroke is one of the major comorbidities of SCD.

A 2016 case study of a sickle cell patient indicated development of acute chest syndrome and failure to modify pain with opioids after the patient had been exposed to the synthetic cannabinoid K2, also known as “Spice”. The patient exhibited delirium and required oxygen support for his first 3 days following hospital admission, after which point the patient admitted to use of K2 at home. The patient’s behavior indicated to the physicians that K2 use was continuing during the hospitalization, and during day 3 acute systolic heart failure was detected. At day 10, the patient was discharged and requested treatment for substance abuse [116]. Use of synthetic drugs labeled cannabinoids share many of the characteristics of intoxication, and also carry risks of dangerous and potentially fatal side effects that include psychosis, seizure, and myocardial infarction [124]. The potency of synthetic cannabinoids derives from their chemical interaction with cannabinoids receptors, for which they are full agonists, whereas THC, the major psychoactive constituent of cannabis, is a partial agonist. These biochemical properties underlie the contrast between synthetic cannabinoids’ apparent toxicity and the lack thereof with THC [33]. The lack of acute toxicity does not mean that THC exposure is without risk.

### 6.7. Cannabis Use in Sickle Cell Trait (SCT)

The use of cannabinoids has been associated with poor health outcomes in patients with SCT, characterized by heterozygosity of the sickle allele. Two case studies have indicated priapism in SCT patients who admitted to using cannabis in the days prior to hospital admission. Notably, these individuals admitted to the use of other substances that contribute to priapism, including alcohol and cocaine in the first patient, and alcohol and tobacco in the second patient [117,118]. Therefore, the effect of cannabis in these studies is confounded by the use of other substances, like many other studies.

## 7. Risks vs. Benefits?

Due to often life-long chronic pain, fear of emerging VOC and rising opioidphobia, SCD patients are more vulnerable to use of cannabis as pain medicine. Cannabis derived cannabinoids have been shown to be safe and well-tolerated in adults across various conditions [106] and, most recently, in SCD [30,111]. Several studies have indicated mild to moderate effectiveness of cannabis in treating pain arising from various disease states [125,126,127], though heterogeneity and low sample sizes mandate replication [107]. Two major considerations for the use of cannabis products in SCD are (i) pregnancy: the use of cannabinoids has been rising in pregnant women [128,129,130,131], and in women with SCD this may be a significant concern due to the discontinuation of hydroxyurea during pregnancy [132]. Early preclinical studies provide mixed evidence for the teratogenicity of cannabinoids, so extreme caution must be taken during pregnancy [133,134]; (ii) depression: Volkow et al. reviewed several studies on adverse health effects of recreational cannabis use and found high confidence in the association between cannabis use and addiction to cannabis, symptoms of chronic bronchitis, motor vehicle accidents, and diminished lifetime achievement, as well as medium confidence in its association with abnormal brain development and depression or anxiety [135]. Recent data indicate the prevalence of depression associated with past month’s cannabis use in adults, thus diligent monitoring for the well-being of patients’ physical and mental health is required [136]. The existence of anxiety, depression and cognitive impairment in SCD warrants the need for a close examination of these features in cannabis users.

### 7.1. Tsunami of Cannabinoids

Innumerable medical cannabis preparations are available from “Dispensaries”, but most of them are not validated for their contents and their effectiveness through regulatory analysis and controlled clinical trials, respectively. Majority of Medical cannabis preparations tested either did not contain the labeled contents or had a small % compared to the labeled amount [137,138]. All medical cannabis preparations are not made equal and may have different cannabinoid content and composition. Therefore, the cannabinoid composition specific to the needs of the underlying pathobiology and symptoms needs to be selected for treatment. Outbreaks of coagulopathy from products marketed as cannabinoids but containing long-acting rodenticide raises life-threatening concerns [139]. Commercially available, mislabeled and adulterated cannabis products pose major health risks [137,138,140]. Therefore, awareness and education of individuals regarding potential harms of the adulterated and unreliable cannabis products needs to be raised and users and healthcare providers need to validate the reliability of the contents.

### 7.2. Major Challenges in the Study and Use of Cannabis in SCD

While many of the aforementioned clinical studies suggest that cannabinoids may be effective therapeutic agents for treating pain, cannabinoid use in the U.S. remains controversial [141,142]. The illicit use of cannabis remains a major concern due in part to racial biases in cannabis sanctions in the U.S. [143], especially for SCD patients that mostly comprise individuals of African descent. As a Schedule I substance, federal law designates cannabis as a drug “with no currently accepted medical use and a high potential for abuse,” but medical cannabis is currently approved in 36 states, Guam, Puerto Rico, US Virgin Islands and District of Columbia [144]. Given the growing legality of medical cannabis use, this substance warrants rigorous study to accurately determine its risks and benefits in SCD.

There is a strong need for randomized, placebo-controlled studies to accurately determine the effects of specific cannabinoids on SCD. Such studies require special attention to not only cannabis dosing and route of administration (e.g., smoked, vaporized, given as an oromucosal spray), but also to the chemical composition of cannabis plants due to existence of variable cannabinoid contents in cannabis plants [13]. Access to cannabis for research purposes remains a major roadblock in the U.S. and many parts of the world despite increasing preclinical evidence suggesting that it may be a valuable strategy for treating otherwise difficult to manage pain, which may be the case in SCD [19,119]. Research funding allocation for cannabis’s safe use in disease-specific manner is needed to prevent the cannabinoid epidemic before it is too late. Given the growing body of evidence supporting the potential benefits of cannabinoids for the treatment of pain in adults, but the lack of randomized, placebo-controlled studies evaluating their use in treating SCD pain, this area of research deems high significance in order to develop more effective therapeutic options requiring more effective management of sickle pain [145,146].

## 8. Conclusions

The complex nature of SCD and its resultant intractable pain make disease management and optimization of analgesia extremely difficult, even with aggressive opioid-based approaches. The paucity of clinical evidence for the effects of cannabis and cannabinoids in SCD is largely due to a lack of rigorously controlled studies; however, the findings from our and other clinical studies indicate positively toward the analgesic potential of cannabis in treating pain arising from SCD and other disease states. The development of a mechanism-based understanding of the effect of cannabinoids on pain, cognitive function, addiction, organ pathology and other comorbidities of SCD is critically needed in pre-clinical and clinical studies. Several major challenges preclude drawing uniform outcomes of cannabinoid use in SCD, which include heterogenous products ranging from medical cannabis to over-the-counter products, as well as unreliable products contaminated with toxic substances, use of other drugs, smaller cohorts in clinical studies, simultaneous use of opioids, stigmatization and variability in presentation, severity and duration of pain. Larger randomized controlled trials on reliable and specific cannabis products are required to disentangle their role as disease-modifying or analgesic agents in the context of SCD without the confounding effect of other substances.

## Figures and Tables

**Table 1:** Cannabis use in sickle cell disease.

Study	Year	Sample Size	Key Finding/Summary	Strengths	Limitations
Abrams et al. [30]	2020	23	Cannabis was safe and significantly improved mood compared to placebo. There was no statistically significant difference, but there was a trend towards reduction in pain interference ratings in cannabis compared to placebo.	Randomized, double-blind, placebo-controlled clinical trial specifically assessing cannabis use for pain in SCD patients.	Small sample size and short duration of treatment.
Curtis et al. [111]	2020	49	SCD patients who use cannabis daily reported more severe pain but had fewer ER visits/hospital admissions compared to non-daily cannabis users with similar pain and disease severity. Daily and non-daily cannabis users reported pain relief as the most common reason for using cannabis.	Cross-sectional survey that examines the link between cannabis use and SCD pain severity and uses propensity score matching to adjust for confounders.	Route and amount of cannabis used was not controlled and presents a confounding factor in this study. Authors report possible selection bias because patients were recruited in clinic visits, and patients with greater disease severity likely present to clinic more often, so they would be more likely to be recruited.
Curtis et al. [112]	2020	75	SCD patients who obtained medical marijuana showed a decrease in admission rates in the 6 months after obtaining medical marijuana, compared to patients who did not obtain it. Patients who obtained medical marijuana had higher baseline use of opioids and illicit cannabis, but neither group demonstrated changes in opioid use.	Retrospective study that evaluates if obtaining medical marijuana is associated with changes in opioids dispensed or in health care utilization.	The method and amount of cannabis used was not controlled. It is unknown if patients who obtained medical marijuana also used illicit marijuana, or used other illicit drugs.
Wilson et al. [113]	2020	291	16.9% of young SCD patients (<25 years) and 21.8% of older SCD patients (≥25 years) used marijuana. Younger patients had lower SCD-related self-care scores and were more likely to have hospital admissions for pain compared to non-users. Older patients using marijuana had more days treating pain at home.	Retrospective study that establishes a link between marijuana use and SCD self-care behaviors and pain management.	Patients may have under-reported illicit marijuana use, as legal medical marijuana was not available during the study period. Information on baseline levels of disease severity was not reported, and presents a confounding factor in the two groups. The data is observational, so a causative relationship between age of marijuana users and self-care/pain management cannot be established.
Wilson et al. [114]	2020	258	24.9% of SCD patients reported substance use. The most commonly used substance was marijuana, with 22.5% of the sample reporting marijuana use, and 15% reporting use in the past 30 days. SCD patients reporting substance use had more stress/distress, higher rates of depression, and poorer quality of life.	Observational study that investigates the relationship between psychosocial/clinical risk factors and substance use in SCD patients.	Patient’s were self-reporting illicit drug use, so may have under-reported. Motivation for substance use and the perceived health benefits of substance use were not investigated. As this is an observational study, it is not possible to establish a causative relationship between substance use and psychosocial/clinical risk factors.
Sinha et al. [21]	2019	15	SCD patients reported increased barriers to opioids for pain management, decreased opioid dosing, physician preoccupation with opiod dosage, and a lack of access to non-opioid therapies, such as marijuana, for pain.	The small sample size allowed for a detailed account of each participant’s point of view in this qualitative study.	This study’s format as a qualitative study with a small, mostly, female sample size makes it difficult to draw conclusions about the greater population of SCD patients. The researchers did not have access to participants’ medical records, so there was no verification of participants’ accounts of opioid experiences.
Curtis et al. [110]	2018	50	Medical marijuana users were more likely to have genotypes associated with more clinically severe SCD, showed a decrease in hospital admissions in the 6 months after obtaining medical marijuana compared to the 6 months prior.	Prospective study that establishes a correlation between medical marijuana use and decreased hospital admissions in SCD patients.	The increased likelihood of patients with more severe SCD to obtain medical marijuana is a confounding variable in this study. The frequency and method of marijuana ingestion were not controlled, making it difficult to establish a causative link between medical marijuana and decreased hospital admissions. Illicit use of other drugs was not evaluated.
Roberts et al. [20]	2018	58 (survey)/57 (urine drug testing)	42% of surveyed SCD patients reported marijuana use, with the majority of users citing medical indications for use. A majority of marijuana users also indicated reduced use of pain medications. On urine drug tests, 18% of patients tested positive for cannabinoids only, 5% tested positive for cannabinoids and cocaine/phencyclidine, and 12% tested positive for cocaine/phencyclidine only.	A survey study that examined the frequency of and reasons for marijuana use in SCD patients.	Patients were self-reporting on illicit marijuana use, so some patients may not have reported their use at all or may have overstated the medicinal benefits of marijuana use. Urine testing was used to estimate marijuana use, so infrequent testing would not account for occasional users. Also, testing was often based on clinician concerns during routine care, so there may have been a sampling bias as patients more likely to show signs of drug abuse may have been more likely to have urine drug testing performed.
Ballas [115]	2017	72 (270 drug screen tests from 72 patients)	SCD patients using cannabis had a higher frequency of VOCs than non-users.	A retrospective study that examines the association between cannabis use and frequency of acute vaso-occlusive crises resulting in hospitalization.	There were statistically significant differences in mixed drug use, frequency of clinic visits, and age of males when comparing the patients who tested positive and negative for cannabinoids. Baseline severity of SCD was not evaluated in the groups of cannabinoid users vs. non-users. These confounders preclude the establishment of a causative link between cannabis use and frequency of VOCs.
Zheng et al. [116]	2016	1	Failure in relieving pain with opioids in a patient with a history of synthetic cannabinoid (K2) use.	Case study that examines the interactions between synthetic cannabinoid use, altered mental status and pain.	Single patient with a history of prior frontal stroke makes it difficult to attribute the patient’s altered mental status solely to the K2 synthetic cannabinoid. However, the toxicity of synthetic drugs raises a concern for safety.
Matta et al. [117]	2014	1	Male Sickle Cell Trait patient with a history of alcohol, tobacco, and cannabis use presented to the emergency department with priapism.	Another case study that examines drug interactions and potential adverse effects associated with cannabis use.	Mixed drug use makes it difficult to extrapolate causation between cannabis and priapism.
Birnbaum, Pinzone [118]	2008	1	Male SCD patient with a history of substance abuse with alcohol, cocaine, and cannabis attempted suicide by quetiapine ingestion, and subsequently developed priapism.	Case study that examines drug interactions and potential adverse effects associated with cannabis use.	Single patient with mixed drug use makes it difficult to extrapolate causation between cannabis and priapism.
Knight-Madden et al. [119]	2006	*n* = 288, *n* = 234 (in 2000 and 2004 respectively)	Among homozygous sickle cell (SS) patients and sickle cell hemoglobin-C disease (SC) patients, the prevalence of smoking marijuana was higher in males and in SC patients. The prevalence of marijuana smoking increased from 2000 to 2004 in both sexes, and marijuana use was not related to SCD complications.	Longitudinal questionnaire-based study that demonstrated high prevalence of marijuana use in SCD patients.	Patients were specifically asked about smoking marijuana, so other methods of use were not represented, and patients were self-reporting on illicit marijuana use, so use may be underreported. The frequency of marijuana use and the type/frequency of SCD complications were not investigated, so motivation for marijuana use remains unclear. Some participants were lost to followup by 2004, while some (*n* = 8) responded only in 2004; it is unclear if these patients were included in the reported results.
Howard et al. [18]	2005	86	36% (*n* = 31) of SCD patients reported using cannabis to relieve pain, anxiety, or depression.	Questionnaire-based survey.	Only 34% of eligible patients completed the questionnaire. Respondents had variable frequency of cannabis use, and there was no quantitative testing to establish baseline pain levels or opioid use prior to cannabis use. Cannabis use may be underreported due to its status as an illicit drug.
Britto et al. [120]	1998	321	Teens with SCD and cystic fibrosis reported significantly less lifetime and current use of marijuana, tobacco, alcohol and fewer other risky behaviors than their healthy peers.	This crossurvey study demonstrates that cannabis use is apparent in SCD teens.	Teens were self-reporting on illicit behaviors, so their responses may not be reliable. Motivation for marijuana use and other risky behaviors were not assessed.
